# Lifetime and Current Self-Harm Thoughts and Behaviors and Their Relationship to Parent and Peer Attachment

**DOI:** 10.1027/0227-5910/a000878

**Published:** 2022-11-02

**Authors:** Julie J. Janssens, Inez Myin-Germeys, Ginette Lafit, Robin Achterhof, Noëmi Hagemann, Karlijn S. F. M. Hermans, Anu P. Hiekkaranta, Aleksandra Lecei, Olivia J. Kirtley

**Affiliations:** ^1^ Center for Contextual Psychiatry, Department of Neuroscience, KU Leuven, Belgium; ^2^ Research Group on Quantitative Psychology and Individual Differences, Department of Psychology, KU Leuven, Belgium; ^3^ Center for Clinical Psychiatry, Department of Neuroscience, KU Leuven, Belgium

**Keywords:** registered report, self-harm, attachment, experience sampling, adolescents

## Abstract

**Abstract.** *Background:* Previous research suggests attachment is a vulnerability factor for self-harm thoughts and behaviors in adults. Yet, few studies have investigated this relationship during adolescence, although adolescence is a critical period for changes in attachment relationships and self-harm onset. Whether and how attachment relates to self-harm thoughts and behaviors as measured in daily life is also unknown. *Aims:* To investigate whether and how paternal, maternal, and peer attachment are associated with lifetime and current adolescent self-harm thoughts and behaviors. Additionally, to examine how different attachment bonds interact in relation to lifetime and current adolescent self-harm thoughts and behaviors. *Method:* Pre-existing data from *N* = 1,913 adolescents of the SIGMA study were used. Attachment and lifetime history of self-harm thoughts and behaviors were measured via retrospective questionnaires. Current self-harm thoughts and behaviors were assessed 10 times per day for 6 days using the experience sampling method (ESM). *Results:* Paternal and maternal attachments were associated with lifetime self-harm thoughts and behaviors and current self-harm thoughts. No significant associations were found between peer attachment and self-harm outcomes. *Limitations:* Some analyses were underpowered. *Conclusion:* Our results highlight the importance of parent–child attachment relationships, which may be intervention targets for prevention and treatment of adolescent self-harm.

Self-harm refers to any act of self-poisoning or self-injury carried out by an individual, irrespective of motivation (National Institute for Health and Care Excellence, 2011). It is among the leading causes of death and injury worldwide (World Health Organization, 2021), and engaging in self-harm significantly increases suicide risk (Hawton et al., 2020). Approximately 90% of people who self-harm began during adolescence (Nock & Prinstein, 2004), with the typical age of onset being around 14 years (Gandhi et al., 2018). To enable early intervention and prevention of self-harm thoughts and behaviors, it is crucial to understand the psychological factors that underpin them.

Previous studies have investigated the role of family functioning in self-harm. The results demonstrated that adolescents reporting reduced family support and excessive behavioral parental control were more likely to report self-harm (Baetens et al., 2015; Palmer et al., 2016). Despite the indication that family functioning may play an important role in self-harm, few studies have substantively examined self-harm in relation to what is potentially the most important aspect of family functioning: attachment.

Bowlby’s attachment theory (1969) contends that secure attachment bonds develop when children generally experience their parents as available, responsive, and attuned. These secure attachment bonds promote the development of stress buffering intrapersonal and interpersonal skills (Bowlby, 1973). Conversely, children with predominantly negative past attachment experiences will develop an insecure parental attachment bond (i.e., lack of trust, poor communication quality, and high interpersonal alienation). Consequently, these children may adopt other, possibly maladaptive, strategies to cope with intense negative emotions, such as self-harm.

Existing studies on attachment and self-harm report conflicting results, and one study found only indirect effects between parental attachment and self-harm via coping strategies (Glazebrook et al., 2016). While some support the importance of maternal and peer bonds (Gandhi et al., 2016), others emphasize the paternal bond (Santens et al., 2018). One potential explanation for these conflicting results is that secure attachment in one domain could protect against insecure attachment in another (Buyse et al., 2011; Mota et al., 2016). Similarly, double-insecure children (i.e., insecurely attached to both parents) may do worse than those with a secure relationship to at least one parent (Kochanska & Kim, 2013). To our knowledge, no published research has investigated the relative and buffering effects of different types of attachment bonds on self-harm within a large, general population sample. Furthermore, most research has focused on individuals from clinical or adult populations (Claes et al., 2016; Glazebrook et al., 2015). As most young people do not present to health services for self-harm (Geulayov et al., 2018; McMahon et al., 2014), research with a general population adolescent sample could increase generalizability and open up new directions for interventions to prevent self-harm in this underserved population.

A further limitation of previous research on attachment and self-harm is that studies have rarely directly investigated whether attachment is differentially associated with thinking (ideating) about self-harm versus engaging in self-harm behaviors. However, contemporary *ideation-to-action* theoretical models of suicidal behavior distinguish between the psychosocial processes associated with self-harm thoughts and those associated with self-harm behaviors (Klonsky & May, 2015; O'Connor & Kirtley, 2018). To this end, the current study builds upon recent research (Zortea et al., 2019) highlighting the utility of the Integrated Motivational–Volitional model (IMV; O'Connor & Kirtley, 2018) for understanding how attachment is related to self-harm thoughts and behaviors. Within the IMV, attachment is posited as a premotivational factor, associated with both thoughts and behaviors (Zortea et al., 2019). To date, however, the relationship between attachment and self-harm in the context of the IMV model has been investigated only in adult samples. Determining whether attachment is differentially related to self-harm thoughts and behaviors in adolescence is of practical importance for early intervention efforts, as most self-harm thoughts and behaviors begin during adolescence.

In addition to investigating whether attachment may differentially relate to self-harm thoughts or behaviors, it is also crucial to measure these thoughts and behaviors where they naturally occur in daily life. Previous research on self-harm in adolescence has relied on retrospective, self-report questionnaires that do not assess the dynamic nature of self-harm. Yet, research assessing self-harm thoughts and behaviors repeatedly within short periods (i.e., hours) is critical to meaningfully capture fluctuations as they occur (Czyz et al., 2019; Glenn et al., 2020). ESM is an intensive longitudinal technique assessing self-reports of behaviors and experiences during daily life (Csikszentmihalyi & Larson, 1987). Participants complete questionnaires on a smartphone, several times a day for multiple days, during their normal everyday life. This technique offers a powerful solution to issues of recall bias and ecological validity by providing us with reliable information about individuals’ self-harm thoughts and behaviors within their real-world context (Myin-Germeys et al., 2018).

In the current study, we aimed to investigate the association between different attachment relationships and self-harm thoughts and behaviors in adolescents. Using pre-existing data from a large, adolescent cohort study (Kirtley et al., 2021), we investigated attachment in relation to both lifetime history and current daily life experiences of self-harm thoughts and behaviors. Specifically, we investigated how mother, father, and peer attachment are associated with lifetime and current self-harm thoughts and behaviors. Additionally, we examined how mother, father, and peer attachment interact with each other in relation to lifetime and current self-harm thoughts and behaviors. We hypothesized that adolescents with higher levels of paternal, maternal, and peer attachment insecurity would be more likely to report lifetime and current self-harm thoughts and behaviors. Furthermore, we predicted that the negative effect of an insecure attachment bond on lifetime and current self-harm thoughts and behaviors would be reduced by security in other attachment bonds.

## Method

### Participants and Recruitment

The data used in this study were drawn from Wave I of the SIGMA study (Kirtley et al., 2021), a large-scale longitudinal study investigating mental health in Flemish adolescents. The original data set includes cross-sectional data from 1,913 adolescents, recruited from the general population via 22 schools across Flanders (Belgium). Schools distributed information letters to potential participants and their caregivers inviting them to participate. Participants were included if they were in their first, third, or fifth year of mainstream secondary education, had an adequate command of the Dutch language, and provided informed consent (from themselves and a caregiver). Most participants within the sample were female (*n* = 1,207; 63%). The age range was 11–20 years (*M* = 13.76 years, *SD* = 1.86 years), with *n* = 1,048 (55%) in their first year, *n* = 424 (22%) in their third year, and *n* = 441 (23%) in their fifth year of mainstream secondary education.

From the full sample (*N* = 1,913), *n* = 1,507 have valid lifetime self-harm data and *n* = 1,788 have valid daily life self-harm data. We used a 10% subsample from both data sets to calculate power and conduct a confirmatory factor analysis (CFA). For the main analyses, samples comprised *n* = 1,272 with valid lifetime self-harm data and at least one observation in the attachment variable and *n* = 1,450 with valid daily life self-harm data and at least one observation in the attachment variable. For more details on this selection procedure, see the supplementary material at https://osf.io/8eu9y/.

### Procedure

The full procedure for the SIGMA project is detailed elsewhere (Kirtley et al., 2021). Data were collected via self-report questionnaires completed during a 100-min in-class testing session, and in daily life using the experience sampling method (ESM), between January 2018 and June 2019. Participants completed a battery of self-report questionnaires via tablets during the testing session. Afterward, participants received a smartphone to complete the ESM questionnaires via the MobileQ application (Meers et al., 2020). Participants were asked to complete 10 ESM questionnaires during a 6-day period. Each day, notifications were randomly distributed in 90-min blocks. The questionnaire consisted of 39–46 items with an estimated completion time of 2–4 min. Participants had 90 s to complete each item. As reimbursement, all participants received a 10-Euro shopping voucher. Participants received no feedback on their compliance or other data during or after the ESM week. Various steps were taken to ensure participant safety and well-being, including provision of support information. For details, see the supplementary material at https://osf.io/8eu9y/. This study received ethical approval from the UZ/KU Leuven Medical Ethics Committee (S61395).

### Measures

The full list of items and details on scoring and construction are included in the supplementary material at https://osf.io/8eu9y/.

#### Attachment

The Dutch version of the Inventory of Parent and Peer Attachment (IPPA; Armsden & Greenberg, 1987; Dutch translation by Noom et al., 1999) is a self-report questionnaire developed to assess the quality of parental and peer attachment. Three dimensions of attachment security are measured: trust, communication, and alienation. A CFA on two subsamples stratified according to the prevalence of lifetime (*n* = 149) and current (*n* = 178) self-harm thoughts and behaviors confirmed a 3-factor structure.

#### Lifetime Self-Harm Thoughts and Behaviors

Four items were used to assess lifetime nonsuicidal and suicidal self-harm thoughts and behaviors, adapted from the Child and Adolescent Self-Harm in Europe study (Madge et al., 2008) questionnaire. All items were translated and back-translated from English to Dutch. Based on these items, three groups were created: 0 = no self-harm thoughts/behaviors, 1 = thoughts only, and 2 = (thoughts and) behaviors.

#### Depression

Depressive symptoms were assessed using the 6-item Depression subscale of the Brief Symptom Inventory (Derogatis & Spencer, 1993), and a sum-score was calculated.

#### Self-Harm Thoughts and Behaviors During Daily Life

In the ESM questionnaire, three self-constructed items were used to assess current self-harm thoughts and behaviors. The three items were branched and presented in a fixed order. The first item was presented at every notification to assess self-harm thoughts. If self-harm thoughts were indicated, one item on self-harm behavior and one item on suicidal intent were followed.

### Missing Data

Due to completely missing data on the self-harm and attachment variable, our final sample sizes were *n* = 1,272 to investigate hypotheses regarding lifetime self-harm and *n* = 1,450 to investigate hypotheses regarding daily life self-harm. Missing attachment data were imputed using multiple imputation using chained equations. For details on missing data analyses, see the supplementary material at https://osf.io/8eu9y/.

### Data Analyses

A model selection procedure was conducted for all models. For further details of the analyses, including power calculations, see https://osf.io/8eu9y/.

#### Attachment and Lifetime Self-Harm Thoughts and Behaviors

To investigate the relationship between attachment (paternal, maternal, and peer) and lifetime self-harm thoughts and behaviors, we first conducted an ordinal logistic regression with self-harm history (0 = no thoughts – no behaviors group, 2 = only thoughts group, and 3 = [thoughts and] behaviors group) as the outcome to test main effects. Paternal, maternal, and peer attachment insecurities were all set simultaneously as the predictor variables in the model. We then investigated each combination of the following interaction terms: Paternal × Maternal × Peer attachment. Given convergence issues during analysis of the stratified subsample (10%; *n* = 149), interaction analyses were exploratory.

#### Attachment and Current Self-Harm Thoughts

The relationships between attachment (paternal, maternal, and peer) and self-harm thoughts in daily life were analyzed using a 2-part mixed-effects model for semicontinuous data (Tom et al., 2016). The model specified a logistic regression for the dichotomous indicator that the outcome is zero or not (0 = having no thoughts or 1 = having thoughts). Then, a standard linear mixed model was estimated for the logarithmic transformation of the nonzero responses (having thoughts with a strength from 1 to 6), allowing for varying intercepts. This model was estimated by specifying a joint distribution that links separate mixed-effects models (i.e., logistic and a linear mixed-effects model for the log-transformed nonzero responses). The two models were linked by covariance between their respective random effects with the results that the two parts of the model were estimated simultaneously (Tooze et al., 2002). Given the general population sample, we expected an excess of zero observations (0 = no self-harm thoughts), and therefore, the 2-part mixed-effects model was especially suitable for analyzing these intensive longitudinal data (Blozis et al., 2020; Farewell et al., 2017).

#### Attachment and Current Self-Harm Behaviors

To investigate the relationship between attachment and self-harm behaviors in daily life (measured using ESM), we conducted a binary logistic mixed-effects regression with the presence of self-harm behavior (0 = no self-harm behavior, 1 = self-harm behavior) as the outcome. We included random intercepts for persons.

In all models, we simultaneously entered paternal, maternal, and peer attachment as predictor variables and controlled for age, gender, and depression assessed at baseline, given their associations with self-harm thoughts and behaviors. To investigate the interaction effect of attachment on current self-harm thoughts and behaviors, we included each combination of the following interaction terms: Paternal × Maternal × Peer Attachment.

#### Power Analysis

A 2-step Monte Carlo simulation approach for power calculation was used, as distributions for the variables of interest could not be found within the existing literature. See supplementary materials for further information at https://osf.io/8eu9y/.

For the hypotheses regarding lifetime self-harm, the results showed that the model performed with sufficient power (.78–1) when investigating the probability of reporting no thoughts versus thoughts. Analysis on the probability of reporting thoughts versus thoughts and behaviors was underpowered (.22–.69).

For analyses on current self-harm, the model was sufficiently powered (1), except for analysis of the relationship between paternal attachment insecurity and self-harm thoughts (the zero part of the model), which was underpowered (.10).

## Deviations From Stage 1

During data analysis, it became clear that we had to deviate slightly from the Stage 1 manuscript. See the supplementary materials at https://osf.io/8eu9y/ for a transparent changes document.

## Results

Descriptive statistics are provided in Table 1. Imputed values were plausible (see supplementary materials at https://osf.io/8eu9y/).

**Table 1:** Descriptive statistics for lifetime (*N* = 1,235) and current (ESM; *N* = 1,210) self-harm sample

Variables	*n* (%)	*M* (*SD*)	*Mdn*	Range
Lifetime samples				
Demographics				
Age (years)		13.86 (1.86)	13	11–19
Gender, % of females		48		
Attachment insecurity				
Paternal		−17.49 (7.39)	−19	−28 to 8
Maternal		−20.46 (6.04)	−22	−28 to 5
Peer		−18.15 (6.11)	−19	−28 to 4
Depressive symptoms		4.60 (4.56)	3	0–20
Lifetime self-harm				
No thoughts/behaviors	726 (59%)			
Thoughts only	248 (20%)			
(Thoughts and) behaviors	261 (21%)			
Current (ESM) sample				
Demographics				
Age (years)		13.77 (1.84)	13	11–19
Gender, % of females		66		
Attachment insecurity				
Paternal		−17.6 (7.24)	−19	−28 to 8
Maternal		−20.44 (6.13)	−22	−28 to 5
Peer		−18.11 (6.05)	−19	−28 to 4
Depressive symptoms		4.61 (4.54)	3	0–20
Current self-harm				
No thoughts/behaviors	522 (43%)			
Thoughts only	390 (32%)			
Thoughts and behaviors	298 (25%)			
Intensity of thoughts^a^	1,210 (100%)	1.25 (0.95)	1	1–7
Intensity of thoughts^b^	688 (57%)	4 (2.16)	4	1–7
Overall compliance		25.88 (12.41)	25	1–59
Number of completed beeps				
*Note*. ^a^For full sample on a scale from 1 to 7. ^b^For thoughts only, and thoughts and behaviors groups on a scale from 1 to 7.				

### Confirmatory Analyses

#### Attachment and Lifetime Self-Harm Thoughts and Behaviors

Higher paternal and maternal attachment insecurities were significantly associated with a greater likelihood of reporting lifetime self-harm thoughts and behaviors, β = 0.03, *SE* = 0.01, *OR* (0*|*1) = 4.11, *OR* (1*|*2) = 15.85, *p* = .002 and β = 0.04, *SE* = 0.01, *OR* (0*|*1) = 4.12, *OR* (1*|*2) = 15.86, *p* < .001, respectively. The association between peer attachment and lifetime self-harm thoughts and behaviors was nonsignificant, β = 0.006, *SE* = 0.01, *OR* (0*|*1) *=* 4*.*09, *OR* (1*|*2) = 15.83, *p* = .06. See Table 2 in the supplementary materials for further results (available at https://osf.io/8eu9y/).

#### Attachment and Current Self-Harm Thoughts and Behaviors

Higher paternal and maternal attachment insecurities were significantly associated with the presence of current self-harm thoughts, β = 0.03, *SE* = 0.01, *OR* (0*|*1) = 0.98, *p* = .02, β = 0.04, *SE* = 0.01, *OR* (0*|*1) = 0.96, *p* < .001, respectively, while peer attachment insecurity was not, β = 0.02, *SE* = 0.01, *OR* (0*|*1) = 0.99, *p* = .16. Associations between attachment (paternal, maternal, and peer) and the intensity of current self-harm thoughts were all nonsignificant, β = −0.002, *SE* = 0.003, *p* = .52, β = 0.002, *SE* = 0.003, *p* = .59, β = −0.006, *SE* = 0.004, *p* = .10, respectively.

Paternal, maternal, or peer attachment insecurities were not significantly associated with the absence/presence of self-harm behaviors, log-odds = −0.001, *SE* = 0.01, *p* = .92; log-odds = 0.03, *SE* = 0.02, *p* = .09; log-odds = −0.02, *SE* = 0.02, *p* = .27, respectively. For further results, see Tables 3 and 4 in the supplementary materials (available at https://osf.io/8eu9y/).

### Exploratory Analyses

Due to space constraints, all exploratory analyses are reported in the supplementary materials at https://osf.io/8eu9y/.

## Discussion

### Confirmatory Analyses

Consistent with previous literature (Gandhi et al., 2016; Santens et al., 2018) and attachment theory (Bowlby, 1969, 1973), we found that adolescents with higher levels of paternal and maternal attachment insecurity were more likely to report lifetime and current self-harm thoughts and lifetime self-harm behaviors. Given the association between attachment insecurity and both lifetime self-harm thoughts and behaviors and current self-harm thoughts, our findings also offer partial support for attachment insecurity as a premotivational variable within the IMV model (O'Connor & Kirtley, 2018), where parent–child attachment relationships may increase vulnerability for self-harm thoughts and behaviors (Zortea et al., 2019). Conversely, self-harm may influence attachment relationships (Ferrey et al., 2016), and this should be investigated in future studies. The findings support prevention and treatment initiatives for adolescent self-harm that restore trust and communication in parent–child relationships, e.g., Attachment-Based Family Therapy (Diamond et al., 2010). However, we found no significant associations between paternal or maternal attachment insecurity and the intensity of current self-harm thoughts or presence of current self-harm behaviors. Since our models of the association between attachment insecurity and lifetime self-harm behaviors, and paternal attachment insecurity and the presence of current self-harm thoughts were underpowered, the results should be interpreted with caution and require replication in larger samples.

Despite the equivocal empirical evidence to date, the lack of an association between peer attachment and self-harm was unexpected (Gandhi et al., 2016). A possible explanation for these results could be the younger average age of the current sample (13.8 years) relative to those of previous studies (≥15.6 years) that found an association between peer attachment insecurity and self-harm (Claes et al., 2010; Jarvi et al., 2013). The effect of peer relationships on psychological outcomes increases with age in the transition to adulthood, where peers become increasingly important (Moretti & Peled, 2004; Wilkinson, 2004); therefore, this relationship may only emerge in older adolescents. Moreover, research has often found only an indirect effect of peer attachment on self-harm via parental attachment or self-esteem (Gandhi et al., 2016; Wilkinson & Walford, 2001).

### Strengths and Limitations

To our knowledge, this is the first study investigating whether adolescents’ attachment insecurity with both parents and peers is associated with self-harm. Given that the current study provides evidence from both retrospective questionnaires and ESM for the relationship between attachment and self-harm, we believe this study contributes to research and practice. Finally, we consider the use of open science practices (the Registered Report article format, open materials, and open code) – still the exception rather than the rule in clinical psychology research (Tackett et al., 2019) – a major strength.

However, several limitations should be discussed. First, researchers have argued that the IPPA assesses parent and peer *relationship quality* rather than attachment (Gandhi et al., 2019); therefore, our study may be more appropriately characterized as investigating relationship quality. Second, some analyses were underpowered. However, the high degree of transparency in our methodology and analysis facilitates future direct replication in higher-powered studies.

### Conclusions

The current study provides evidence for the association of both paternal attachment and maternal attachment with lifetime and current self-harm thoughts and lifetime self-harm behaviors, during adolescence, and provides support for attachment as a premotivational variable within the IMV model. Parent–child attachment relationships may be promising intervention targets for the prevention and treatment of adolescent self-harm. Future research should further investigate the buffering effects of different types of attachment bonds on self-harm.

## Notes

**Publication Ethics:** This study received ethical approval from the UZ/KU Leuven Medical Ethics Committee (S61395).

**Open Data:** Details on selection procedure, data access confirmation, overview of hypotheses and analysis plan (postregistration), confirmatory factor analysis, group comparison analyses, draft/final R codes, and output files of all main and exploratory analyses with and without the suicidal ideation item of the BSI, full list and details on scoring and construction of all items and questionnaires, cover letter, details on missing data analysis and quality check of imputed values, details on power calculation, transparent changes document, and results of all exploratory analyses are available at https://osf.io/8eu9y/ (Janssens et al., 2022).

The Stage 1 version of the manuscript is also available at https://osf.io/8eu9y/ (Janssens et al., 2022).

## References

[c1] Armsden, G. C., & Greenberg, M. T. (1987). The inventory of parent and peer attachment: Individual differences and their relationship to psychological well-being in adolescence. *Journal of Youth and Adolescence*, *16*(5), 427–454. 10.1007/BF02202939 24277469

[c2] Baetens, I., Andrews, T., Claes, L., & Martin, G. (2015). The association between family functioning and NSSI in adolescence: The mediating role of depressive symptoms. *Family Science*, *6*(1), 330–337. 10.1080/19424620.2015.1056917

[c3] Blozis, S. A., McTernan, M., Harring, J. R., & Zheng, Q. (2020). Two-part mixed-effects location scale models. *Behavior Research Methods*, *52*(5), 1836–1847. 10.3758/s13428-020-01359-7 32043225

[c4] Bowlby, J. (1969). *Attachment and loss: Vol. 1 Attachment* (2^nd^ ed.). Basic Books.

[c5] Bowlby, J. (1973). *Attachment and loss: Vol 2: Separation: Anxiety and anger*. The Hogarth Press and the Institute of Psycho-Analysis.

[c6] Buyse, E., Verschueren, K., & Doumen, S. (2011). Preschoolers' attachment to mother and risk for adjustment problems in kindergarten: Can teachers make a difference? *Social Development*, *20*(1), 33–50. 10.1111/j.1467-9507.2009.00555.x

[c7] Claes, L., De Raedt, R., Van de Walle, M., & Bosmans, G. (2016). Attentional bias moderates the link between attachment-related expectations and non-suicidal self-injury. *Cognitive Therapy and Research*, *40*(4), 540–548. 10.1007/s10608-016-9761-5

[c8] Claes, L., Houben, A., Vandereycken, W., Bijttebier, P., & Muehlenkamp, J. (2010). Brief report: The association between non-suicidal self-injury, self-concept and acquaintance with self-injurious peers in a sample of adolescents. *Journal of Adolescence*, *33*(5), 775–778. 10.1016/j.adolescence.2009.10.012 19910041

[c9] Csikszentmihalyi, M., & Larson, R. (1987). Validity and reliability of the experience-sampling method. *The Journal of Nervous and Mental Disease*, *175*(9), 526–536. 10.1097/00005053-198709000-00004 3655778

[c10] Czyz, E. K., Glenn, C. R., Busby, D., & King, C. A. (2019). Daily patterns in nonsuicidal self-injury and coping among recently hospitalized youth at risk for suicide. *Psychiatry Research*, *281*. Article 112588. 10.1016/j.psychres.2019.112588 PMC6890202 31629299

[c11] Derogatis, L. R., & Spencer, P. M. (1993). *Brief symptom inventory: BSI*. Pearson.

[c12] Diamond, G. S., Wintersteen, M. B., Brown, G. K., Diamond, G. M., Gallop, R., Shelef, K., & Levy, S. (2010). Attachment-based family therapy for adolescents with suicidal ideation: A randomized controlled trial. *Journal of the American Academy of Child & Adolescent Psychiatry*, *49*(2), 122–131. 20215934 10.1097/00004583-201002000-00006

[c13] Farewell, V. T., Long, D. L., Tom, B. D. M., Yiu, S., & Su, L. (2017). Two-part and related regression models for longitudinal data. *Annual Review of Statistics and its Application*, *4*, 283–315. 10.1146/annurev-statistics-060116-054131 PMC5590716 28890906

[c14] Ferrey, A. E., Hughes, N. D., Simkin, S., Locock, L., Stewart, A., Kapur, N., Gunnell, D., & Hawton, K. (2016). The impact of self-harm by young people on parents and families: A qualitative study. *BMJ Open*, *6*(1). Article e009631. 10.1136/bmjopen-2015-009631 PMC4716183 26739734

[c15] Gandhi, A., Claes, L., Bosmans, G., Baetens, I., Wilderjans, T. F., Maitra, S., Kiekens, G., & Luyckx, K. (2016). Non-suicidal self-injury and adolescents attachment with peers and mother: The mediating role of identity synthesis and confusion. *Journal of Child and Family Studies*, *25*(6), 1735–1745. 10.1007/s10826-015-0350-0

[c16] Gandhi, A., Luyckx, K., Baetens, I., Kiekens, G., Sleuwaegen, E., Berens, A., Maitra, S., & Claes, L. (2018). Age of onset of non-suicidal self-injury in Dutch-speaking adolescents and emerging adults: An event history analysis of pooled data. *Comprehensive Psychiatry*, *80*, 170–178. 10.1016/j.comppsych.2017.10.007 29121554

[c17] Gandhi, A., Luyckx, K., Molenberghs, G., Baetens, I., Goossens, L., Maitra, S., & Claes, L. (2019). Maternal and peer attachment, identity formation, and non-suicidal self-injury: A longitudinal mediation study. *Child and Adolescent Psychiatry and Mental Health*, *13*(1), 1–11. 10.1186/s13034-019-0267-2 30675177 PMC6339302

[c18] Geulayov, G., Casey, D., McDonald, K. C., Foster, P., Pritchard, K., Wells, C., Clements, C., Kapur, N., Ness, J., Waters, K., & Hawton, K. (2018). Incidence of suicide, hospital-presenting non-fatal self-harm, and community-occurring non-fatal self-harm in adolescents in England (the iceberg model of self-harm): A retrospective study. *The Lancet Psychiatry*, *5*(2), 167–174. 10.1016/S2215-0366(17)30478-9 29246453

[c19] Glazebrook, K., Townsend, E., & Sayal, K. (2015). The role of attachment style in predicting repetition of adolescent self-harm: A longitudinal study. *Suicide and Life-Threatening Behavior*, *45*(6), 664–678. 10.1111/sltb.12159 25845416 PMC6680138

[c20] Glazebrook, K., Townsend, E., & Sayal, K. (2016). Do coping strategies mediate the relationship between parental attachment and self-harm in young people?. *Archives of Suicide Research*, *20*(2), 205–218. 10.1080/13811118.2015.1004495 26699201

[c21] Glenn, C. R., Kleiman, E. M., Kearns, J. C., Santee, A. C., Esposito, E. C., Conwell, Y., & Alpert-Gillis, L. J. (2020). Feasibility and acceptability of ecological momentary assessment with high-risk suicidal adolescents following acute psychiatric care. *Journal of Clinical Child & Adolescent Psychology*, *51*(1), 32–48. 10.1080/15374416.2020.1741377 32239986

[c22] Hawton, K., Bale, L., Brand, F., Townsend, E., Ness, J., Waters, K., Clements, C., & Geulayov, G. (2020). Mortality in children and adolescents following presentation to hospital after non-fatal self-harm in the Multicentre Study of Self-harm: A prospective observational cohort study. *The Lancet Child & Adolescent Health*, *4*(2), 111–120. 10.1016/S2352-4642(19)30373-6 31926769

[c23] Janssens, J., Myin-Germeys, I., Lafit, G., Achterhof, R., Hageman, N., Hermans, K., Hiekkaranta, A. P., Lecei, A., & Kirtley, O. (2022). *Lifetime and current self-harm thoughts and behaviours and their relationship to parent and peer attachment*. [Data set]. https://osf.io/8eu9y/10.1027/0227-5910/a000878 PMC10556547 36321256

[c24] Jarvi, S., Jackson, B., Swenson, L., & Crawford, H. (2013). The impact of social contagion on non-suicidal self-injury: A review of the literature. *Archives of Suicide Research*, *17*(1), 1–19. 10.1080/13811118.2013.748404 23387399

[c25] Kirtley, O., Achterhof, R., Hagemann, N., Hermans, K., Hiekkaranta, A. P., Lecei, A., Boets, B., Henquet, C., Kasanova, Z., Schneider, M., Van Winkel, R., Reininghaus, U., Viechtbauer, W., & Myin-Germeys, I. (2021). Initial cohort characteristics and protocol for SIGMA: An accelerated longitudinal study of environmental factors, inter-and intrapersonal processes, and mental health in adolescence. *PsyArXiv Preprints*. 10.31234/osf.io/jp2fk

[c26] Klonsky, E. D., & May, A. M. (2015). The three-step theory (3ST): A new theory of suicide rooted in the “ideation-to-action” framework. *International Journal of Cognitive Therapy*, *8*(2), 114–129. 10.1521/ijct.2015.8.2.114

[c27] Kochanska, G., & Kim, S. (2013). Early attachment organization with both parents and future behavior problems: From infancy to middle childhood. *Child Development*, *84*(1), 283–296. 10.1111/j.1467-8624.2012.01852.x 23005703 PMC3530645

[c28] Madge, N., Hewitt, A., Hawton, K., de Wilde, E. J., Corcoran, P., Fekete, S., van Heeringen, K., De Leo, D., & Ystgaard, M. (2008). Deliberate self-harm within an international community sample of young people: Comparative findings from the Child & Adolescent Self-harm in Europe (CASE) study. *Journal of Child Psychology and Psychiatry*, *49*(6), 667–677. 10.1111/j.1469-7610.2008.01879.x 18341543

[c29] McMahon, E. M., Keeley, H., Cannon, M., Arensman, E., Perry, I. J., Clarke, M., Chambers, D., & Corcoran, P. (2014). The iceberg of suicide and self-harm in Irish adolescents: A population-based study. *Social Psychiatry and Psychiatric Epidemiology*, *49*(12), 1929–1935. 10.1007/s00127-014-0907-z 24929354

[c30] Meers, K., Dejonckheere, E., Kalokerinos, E. K., Rummens, K., & Kuppens, P. (2020). mobileQ: A free user-friendly application for collecting experience sampling data. *Behavior Research Methods*, *52*, 1510–1515. 10.3758/s13428-019-01330-1 31898294

[c31] Moretti, M. M., & Peled, M. (2004). Adolescent-parent attachment: Bonds that support healthy development. *Paediatrics & Child Health*, *9*(8), 551–555. 10.1093/pch/9.8.551 19680483 PMC2724162

[c32] Mota, C. P., Costa, M., & Matos, P. M. (2016). Resilience and deviant behavior among institutionalized adolescents: The relationship with significant adults. *Child and Adolescent Social Work Journal*, *33*(4), 313–325. 10.1007/s10560-015-0429-x

[c33] Myin-Germeys, I., Kasanova, Z., Vaessen, T., Vachon, H., Kirtley, O., Viechtbauer, W., & Reininghaus, U. (2018). Experience sampling methodology in mental health research: New insights and technical developments. *World Psychiatry*, *17*(2), 123–132. 10.1002/wps.20513 29856567 PMC5980621

[c34] National Institute for Health and Care Excellence. (2011). *Self-harm (NICE guideline*). https://www.nice.org.uk/guidance

[c35] Nock, M. K., & Prinstein, M. J. (2004). A functional approach to the assessment of self-mutilative behavior. *Journal of Consulting and Clinical Psychology*, *72*(5), 885–890. 10.1037/0022-006X.72.5.885 15482046

[c36] Noom, M. J., Deković, M., & Meeus, W. H. (1999). Autonomy, attachment and psychosocial adjustment during adolescence: A double-edged sword?. *Journal of Adolescence*, *22*(6), 771–783. 10.1006/jado.1999.0269 10579889

[c37] O'Connor, R. C., & Kirtley, O. J. (2018). The integrated motivational–volitional model of suicidal behaviour. *Philosophical Transactions of the Royal Society B: Biological Sciences*, *373*(1754). Article 20170268. 10.1098/rstb.2017.0268 PMC6053985 30012735

[c38] Palmer, E., Welsh, P., & Tiffin, P. A. (2016). Perceptions of family functioning in adolescents who self-harm. *Journal of Family Therapy*, *38*(2), 257–273. 10.1111/1467-6427.12069

[c39] Santens, T., Claes, L., Diamond, G. S., & Bosmans, G. (2018). Depressive symptoms and self-harm among youngsters referred to child welfare: The role of trust in caregiver support and communication. *Child Abuse & Neglect*, *77*, 155–167. 10.1016/j.chiabu.2018.01.001 29353719

[c40] Tackett, J. L., Brandes, C. M., & Reardon, K. W. (2019). Leveraging the Open Science Framework in clinical psychological assessment research. *Psychological Assessment*, *31*(12), 1386. 10.1037/pas0000583 30869959

[c41] Tom, B. D., Su, L., & Farewell, V. T. (2016). A corrected formulation for marginal inference derived from two-part mixed models for longitudinal semi-continuous data. *Statistical Methods in Medical Research*, *25*(5), 2014–2020. 10.1177/0962280213509798 24201470 PMC5051603

[c42] Tooze, J. A., Grunwald, G. K., & Jones, R. H. (2002). Analysis of repeated measures data with clumping at zero. *Statistical Methods in Medical Research*, *11*(4), 341–355. 10.1191/0962280202sm291ra 12197301

[c43] Wilkinson, R. B. (2004). The role of parental and peer attachment in the psychological health and self-esteem of adolescents. *Journal of Youth and Adolescence*, *33*(6), 479–493. 10.1023/B:JOYO.0000048063.59425.20

[c44] Wilkinson, R. B., & Walford, W. A. (2001). Attachment and personality in the psychological health of adolescents. *Personality and Individual Differences*, *31*(4), 473–484. 10.1016/S0191-8869(00)00151-3

[c45] World Health Organization. (2021). * Suicide worldwide in 2019: Global health estimates*. https://www.who.int/publications/i/item/9789240026643

[c46] Zortea, T. C., Gray, C. M., & O’Connor, R. C. (2019). Perceptions of past parenting and adult attachment as vulnerability factors for suicidal ideation in the context of the integrated motivational–volitional model of suicidal behavior. *Suicide and Life-Threatening Behavior*, *50*(2), 515–533. 10.1111/sltb.12606 31763711

